# A clinical assessment of three-dimensional-printed liver model navigation for thrice or more repeated hepatectomy based on a conversation analysis

**DOI:** 10.1007/s00595-024-02835-9

**Published:** 2024-04-12

**Authors:** Tsuyoshi Igami, Akihiro Maehigashi, Yoshihiko Nakamura, Yuichiro Hayashi, Masahiro Oda, Yukihiro Yokoyama, Takashi Mizuno, Junpei Yamaguchi, Shunsuke Onoe, Masaki Sunagawa, Nobuyuki Watanabe, Taisuke Baba, Shoji Kawakatsu, Kensaku Mori, Kazuhisa Miwa, Tomoki Ebata

**Affiliations:** 1 Division of Surgical Oncology, Department of Surgery, Nagoya University Graduate School of Medicine, 65 Tsurumai-Cho, Showa-Ku, Nagoya, 466-8550 Japan; 2 Center for Research and Development in Admissions, Shizuoka University, Shizuoka, Japan; 3 Division of Computer Science and Engineering, Department of Engineering for Innovation, National Institute of Technology, Tomakomai College, Tomakomai, Japan; 4 Information Strategy Office, Information and Communications, Nagoya University, Nagoya, Japan; 5 Graduate School of Informatics, Department of Intelligent Systems, Nagoya University, Nagoya, Japan; 6 Graduate School of Informatics, Department of Cognitive and Psychological Sciences, Nagoya University, Nagoya, Japan

**Keywords:** Three-dimensional-printed liver model, Hepatectomy repeated three or more times, Clinical assessment of navigation surgery, Navigation surgery, Conversation analysis

## Abstract

**Purposes:**

We performed a conversation analysis of the speech conducted among the surgical team during three-dimensional (3D)-printed liver model navigation for thrice or more repeated hepatectomy (TMRH).

**Methods:**

Seventeen patients underwent 3D-printed liver navigation surgery for TMRH. After transcription of the utterances recorded during surgery, the transcribed utterances were coded by the utterer, utterance object, utterance content, sensor, and surgical process during conversation. We then analyzed the utterances and clarified the association between the surgical process and conversation through the intraoperative reference of the 3D-printed liver.

**Results:**

In total, 130 conversations including 1648 segments were recorded. Utterance coding showed that the operator/assistant, 3D-printed liver/real liver, fact check (F)/plan check (Pc), visual check/tactile check, and confirmation of planned resection or preservation target (T)/confirmation of planned or ongoing resection line (L) accounted for 791/857, 885/763, 1148/500, 1208/440, and 1304/344 segments, respectively. The utterance’s proportions of assistants, F, F of T on 3D-printed liver, F of T on real liver, and Pc of L on 3D-printed liver were significantly higher during non-expert surgeries than during expert surgeries. Confirming the surgical process with both 3D-printed liver and real liver and performing planning using a 3D-printed liver facilitates the safe implementation of TMRH, regardless of the surgeon’s experience.

**Conclusions:**

The present study, using a unique conversation analysis, provided the first evidence for the clinical value of 3D-printed liver for TMRH for anatomical guidance of non-expert surgeons.

## Introduction

Repeated hepatectomy for patients with hepatocellular carcinoma or colorectal liver metastasis is widely accepted to prolong the survival after primary hepatectomy [1–7]. In repeated hepatectomy, precise inspection by intraoperative ultrasonography is quite important but is technically demanding due to intrahepatic air introduced by injury of the liver surface during adhesiotomy and/or changes in the liver shape after a previous hepatectomy [7–10]. In particular, this technical difficulty is remarkable when three or more repeated hepatectomies are performed. Recently, some authors have reported the application of 3D printing technology in living-donor liver transplantation [11, 12]. In addition, we used this cutting-edge technology in practice, reporting its feasibility and utility in two challenging situations: hepatectomy for small, ultrasonographically invisible tumors and minor hepatectomy after liver partition along the right portal fissure [13, 14]. In all of these prior studies, the clinical significance of navigation surgery was evaluated exclusively by the accuracy of navigation, i.e., the anatomical landmark or orienting ability of the navigation system.

In the field of psychology, it is believed that cognitive activities through interactions between the internal human mind and the external environment and decision-making based on information and constraints provided by external resources are important for solving problems, and a conversation analysis is useful for analyzing such cognitive performance [15–18]. In addition, in our preliminary experience, analyzing conversations before, during, and after utilization of three-dimensional (3D)-printed liver models revealed that utilization of such models enhanced the construction of elaborate internal mental models of patients’ livers, mental simulation of liver resections, and construction of shared mental models of patients’ livers among doctors [19].

The present study used an ethnographic analysis to conduct a new clinical assessment of 3D-printed liver model navigation based on a conversation analysis during thrice or more repeated hepatectomy.

## Patients ad methods

### Patients

We applied 3D printing of the liver to thrice or more repeated hepatectomy in 17 cases at Nagoya University Hospital between 2015 and 2022. There were 15 males and 2 females. This study was approved by the Human Research Review Committee of Nagoya University Hospital (approval number 2020–0177).

### 3D printing methods

**Fig. 1:**
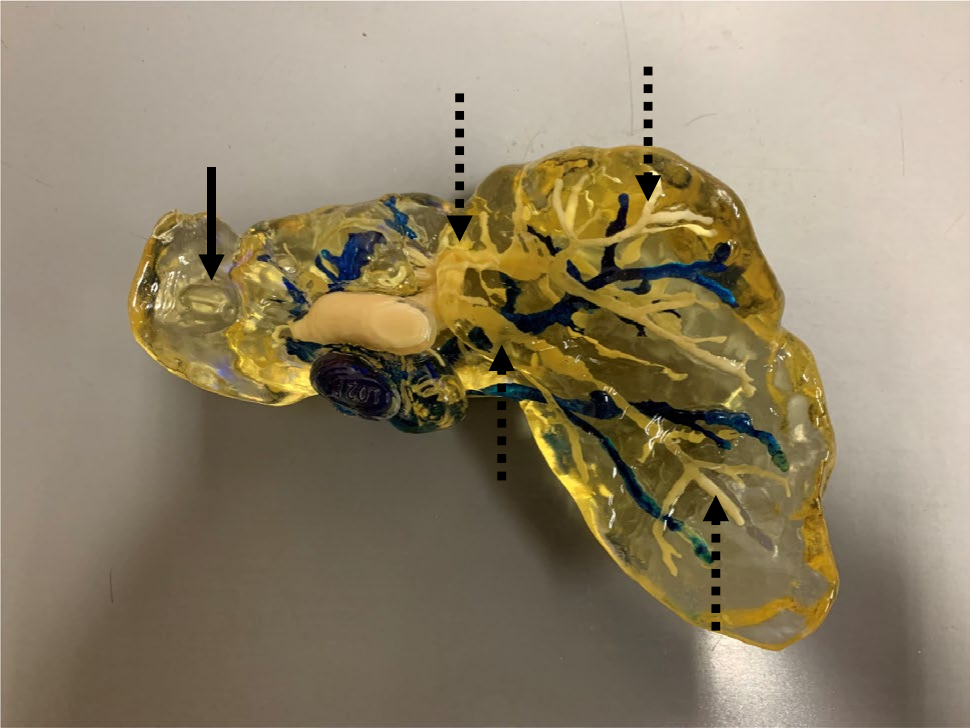
3D-printed liver model. After handwork, a whitish tumor (black solid arrow), whitish 3D-printed portal veins (black broken arrows), and colored 3D-printed hepatic veins with a 3D-printed inferior vena cava were well visualized in the 3D-printed liver model

As described in our previous report [13, 14], the details of the 3D printing methods are as follows: Anatomical structures (livers, tumors, portal veins, and hepatic veins) were digitally segmented with the original software program “PLUTO” [20], which was developed by the Graduate School of Information Science of Nagoya University (Nagoya, Japan) from multidetector row computed tomography (MDCT) images. Subsequently, digital segmentation data were arranged such that the mold material would remain in the 3D-printed portal veins and be removed from the 3D-printed hepatic veins. The final digital segmentation data were converted to stereolithography (STL) files by an additional function of “PLUTO,” which utilized the “Marching Cubes” procedure [21]. The final STL files generated via digital preparation were imported into a 3D printer (AGILISTA-3100; Keyence Co., Osaka, Japan), and a 3D-printed liver model was produced. The 3D-printed liver model was printed at 70% of the size of a life-sized liver. The material used for printing was a rigid acrylic resin, and the mold and support materials were water-soluble acrylic resin. Immediately after 3D printing, the surface of the 3D-printed liver model was covered with support material. The support material was washed away, and the mold material was removed from the 3D-printed hepatic using an ultrasonic washing machine. The surface of the 3D-printed liver model was abraded and coated with urethane resin. After natural drying, the 3D-printed hepatic veins were colored by injecting a dye (Indigo Carmine, Daiichi Sankyo Co., Tokyo, Japan). The 3D-printed portal veins and tumor were whitish because the mold material remained (Fig. 1). All of the procedures were performed manually after 3D printing.

### Utilization of 3D-printed livers

The 3D-printed liver model was utilized for thrice or more repeated hepatectomy, hepatectomy after hepatobiliary resection and/or pancreatoduodenectomy, hepatectomy for invisible tumors by ultrasonography, and small hepatectomy after liver partition.

About a week before surgery, the 3D-printed liver model reached the surgical teams, and a preoperative conference regarding the surgical plan was convened using the 3D-printed liver model.

Just before surgery, the 3D-printed liver model was packed into a sterilized nylon bag using a vacuum compressor [13, 14]. If the 3D-printed liver model broke during surgery, this package prevented even a small fragment from disappearing into the abdominal cavity.

In the study patients, the 3D-printed liver model was utilized at the time of drawing the cut line on the liver surface and at the interval of Pringle’s maneuver. In addition, at any point of dithering and/or freezing with regard to the decision concerning the cut line and/or cut direction of parenchyma, the 3D-printed liver model was utilized. According to the 3D-printed liver model navigation with conversation, the surgeons understood the anatomical relationship and made decisions regarding the surgical plan and procedure.

In this series, intraoperative ultrasound was used only at the time of liver screening to identify tumors other than those preoperatively diagnosed before hepatectomy.

### Conversation analyses

**Fig. 2:**
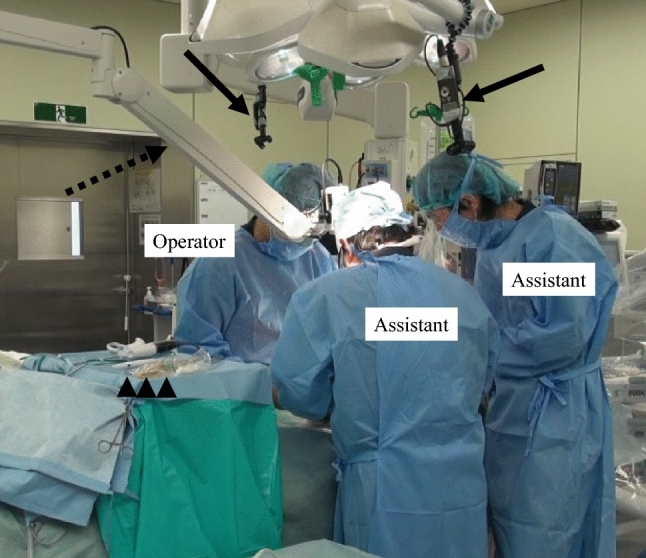
Video and voice recording during surgery. This picture is a snapshot of the recorded video using a fixed video camera with a voice recorder during surgery (Case 14 in Table 1). Two voice recorders (black solid arrows) and one video camera with a voice recorder (black broken arrow) were used to record the video and voice during the surgery. After surgery, all utterances through the intraoperative reference of the 3D-printed liver model (black arrowheads) were transcribed

**Fig. 3:**
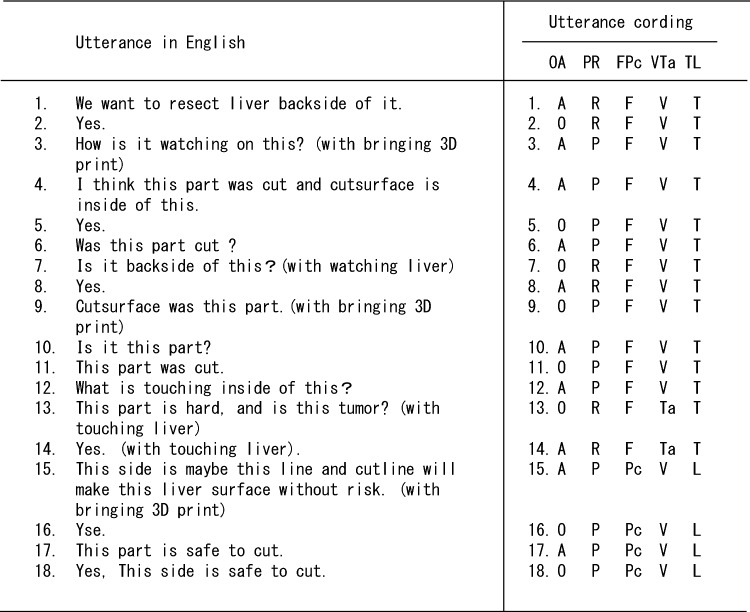
Utterance coding before, during, and after utilization of the 3D-printed liver model. After transcription in Japanese, all utterances through intraoperative reference of the 3D-printed model were coded according to the following five factors: utterer (operator or assistants), utterance object (3D-printed liver model or the real liver), utterance content (fact check or plan check), sense used during the conversation (visual check or tactile check), and surgical process during the conversation (confirmation of the planned resection and/or preservation target or confirmation of the planned and/or ongoing resection line). In addition, all utterances in Japanese were translated into English for clarity. OA, operator or assistants; O, operator; A, assistant; PR, 3D-printed liver model or real liver; P, 3D-printed liver model; R, real liver; FPc, fact check or plan check; F, fact check; Pc, plan check; VTa, visual check or tactile check; V, visual check; Ta, tactile check; TL, confirmation of the planned resection and/or preservation target or confirmation of the planned and/or ongoing resection line; T, confirmation of the planned resection and/or preservation target; L, confirmation of the planned and/or ongoing resection line

According to the findings described in our previous proceeding sheets [19], all spoken words and actions of the surgeon and assistant during surgery were recorded with video and voice recorders (Fig. 2) [21]. One utterance was defined as assertiveness in a conversation and/or delimiter of action. Confirmation of the location, length, and size of the anatomical structure and/or tumor in the liver was defined as a “fact check”. Confirmation of the resection target (e.g., cutting this artery, preserving this area, dividing this area, etc.) and the resection line in the liver (e.g., cutting a certain line, cutting along a certain line, dividing the parenchyma in a certain dimension, etc.) were defined as “plan checks”. Inspecting the real liver by touch (i.e., hard or soft) and touching the tumor through the liver were defined as “tactile checks”. Distance (e.g., 3 cm, a certain interval, or the length from one part to another) and the positional relationship (e.g., above the artery, inside a certain part, on the surface of the liver, etc.) were defined as “visual checks” (Fig. 3).

**Table 1:** Clinical characteristics of patients who underwent thrice or more repeated hepatectomy using a 3D print of a liver

	Age (years)	Sex	Disease	Present hepatectomy^a^	Interval time^b^ (days)	Hepatectomy history^a^				Surgeon	Operative time (minutes)	Blood loss (mL)
						1st	2nd	3rd	4th			
1	80	M	HCC	H2′	140	H5′	Lap-H5′67′			Expert	142	100
2	58	F	META-G	H6′/6′/7′8′	2493	GBR	Lap-H5′			Expert	245	662
3	69	M	META	H1′/5′	327	H23/4′8′	H1″			Expert	434	2456
4	65	M	META	H67	455	Lap-H6′	Lap-H2′/S6′			Expert	444	1830
5	76	M	HCC	H7′	296	Lap-H5′6	H7′			Expert	229	500
6	56	M	HCC	H5′	641	Lap-H2′3′	H8′			Non-expert	166	62
7	60	M	HCC	H3′/6′	149	Lap-H8′	H8′/6′			Non-expert	275	2684
8	64	M	HCC	H3	244	Lap-H8′	H1′			Non-expert	192	254
9	67	M	META	H45678 + Dia	1547	GBR	H5′8′			Non-expert	616	607
10	71	F	HCC	H5′/8′	2618	Lap-H8′	Lap-H23			Non-expert	336	578
11	71	M	HCC	H3′	826	Lap-H6′	H5′/H7′			Non-expert	160	353
12	80	M	HCC	H8′	371	H5′	Lap-H5′67′	H2′		Expert	336	1617
13	79	M	HCC	H3′/7′8′	823	Lap-H5bc6	H7′	H7′		Non-expert	307	552
14	60	M	HCC	H6′	173	Lap-H8′	H8′/6′	H3′/6′		Non-expert	329	1354
15	70	M	META	H8 + IVC	1638	Lap-H6′	Lap-H2′/S6′	H67		Non-expert	755	14,033
16	79	M	HCC	H5678	246	Lap-H5′6	H7′	H7′	H3′/7′8′	Non-expert	586	6134
17	61	M	HCC	H3′	315	Lap-H8′	H8′/6′	H3′/6′	H6′	Non-expert	320	221

M, male; F, female; HCC, hepatocellular carcinoma; META, liver metastasis from colorectal carcinoma; META-G, liver metastasis from gallbladder carcinoma; Dia, combined resection of the diaphragm and reconstruction using the flap of the rectus abdominis with vascular pedicle; IVC, resection of intravenous tumor in the infra vena cava using extracorporeal circulation; GBR, gallbladder bed resection; Lap, laparoscopic hepatectomy; Expert, an expert surgeon of hepatobiliary surgery; Non-expert, a surgeon permitted to perform hepatobiliary surgery under an expert’s guidance

^a^ Terminology of hepatectomy according to the New World Terminology[22]

^b^ Interval time between the present and latest hepatectomies

After transcription, all utterances through intraoperative reference of the 3D-printed model were coded according to the following five factors: utterer (operator or assistants), utterance object (the object referenced in the utterance, i.e., the 3D-printed liver model or the real liver), utterance content (fact check or plan check), the sense used during conversation (visual or tactile check), and the surgical process used during conversation (confirmation of the planned resection and/or preservation target or confirmation of the planned and/or ongoing resection line) (Fig. 3). These classifications were performed by a surgeon (T. I.) and psychologist (A. M.). We analyzed utterance coding and clarified the association between surgical progress and conversation through intraoperative reference of 3D-printed liver models. In addition, we compared utterances during surgery by an expert surgeon, who is a specialist in hepatobiliary surgery and a board-certified instructor and/or a board-certified surgeon of the Japanese Society of Hepato-Biliary-Pancreatic Surgery, and those by a non-expert surgeon, who is not a board-certified surgeon of the Japanese Society of Hepato-Biliary-Pancreatic Surgery but can perform hepatobiliary surgery under the guidance of an expert surgeon. Because we only analyzed conversations between surgeons in this study, other conversations between surgeons, nursing staff, and anesthesiologists were excluded from this analysis.

### Statistical analyses

Continuous variables, expressed as mean ± standard deviation (SD) unless specified otherwise, were compared using the Mann–Whitney *U*-test. Categorical variables were analyzed using the *χ*^2^ test or Fisher’s exact test, as appropriate. All tests were two-sided, and *p* < 0.05 was considered to indicate statistical significance. Statistical calculations were performed using the IBM SPSS Statistics software program for Windows, version 22 (IBM Co., New York, USA.).

## Results

### Clinical characteristics

The clinical characteristics of these 17 patients are presented in Table 1. The terminology for hepatectomy was described according to the New World Terminology [22]. Eleven patients (hepatocellular carcinoma, *n* = 8; metastatic liver tumor, *n* = 3) had undergone thrice repeated hepatectomy. Of these 11 patients, 4 (hepatocellular carcinoma, *n* = 3; metastatic liver tumor, *n* = 1) underwent fourth time’s repeated hepatectomy following thrice repeated hepatectomy, and 2 (hepatocellular carcinoma, *n* = 2) underwent fifth time’s repeated hepatectomy following the fourth time’s repeated hepatectomy. The mean interval between the present and latest hepatectomies was 782 ± 780 (range, 140–2618) days.

There were no significant differences in the mean operative time between cases with thrice repeated hepatectomy (294 ± 142 min; range, 142–616 min) and that of the fourth or fifth time’s repeated hepatectomies (439 ± 171 min; range, 307–755 min) (*p* = 0.101). There were no significant differences in the mean blood loss between the thrice repeated hepatectomy (917 ± 901 mL; range, 62–2684 mL) and fourth- or fifth time’s repeated hepatectomies (3985 ± 4900 mL; range, 221–14,033 mL) (*p* = 0.778).

Comparison according to the surgeon’s experience showed that there were no significant differences between the mean operative time for surgeries performed by experts (305 ± 121 min; range, 142–444 min) and those performed by non-experts (367 ± 198 min; range, 160–755 min) (*p* = 0.495). There were also no significant differences in the mean blood loss between surgeries performed by experts (1194 ± 910 mL; range, 100–2456 mL) and those performed by non-experts (2439 ± 4235 mL; range, 62–14,033 mL) (*p* = 0.494).

Pathological margin-free resection (R0) was achieved in 16 patients, and 1 patient who underwent right trisectionectomy with concomitant resection of the diaphragm underwent R1 resection.

### Utterance coding

Utterance coding through intraoperative reference of the 3D-printed liver in the 17 study patients is shown in Table 2. Over a total of 130 conversations, which included 1648 segments, there were 791 segments uttered by the operator and 857 by the assistant. Regarding the utterance object, 885 segments were utterances associated with 3D-printed models, and 763 were utterances associated with the real liver. Regarding the utterance content, 1148 and 500 segments were classified as a fact check and plan check, respectively. Regarding the senses used during conversation, visual and tactile checks were utilized in 1208 and 440 segments, respectively. Regarding the surgical process during conversation, 1304 segments were associated with confirmation of the planned resection and/or preservation target, and 344 were associated with confirmation of the planned and/or ongoing resection line.

**Table 2:** Utterance coding through intraoperative reference of a 3D print of a liver for thrice or more repeated hepatectomy

	Conversation	Utterance cording				
		Utterer	Object	Content	Sense	Process
		(Operator:Assistants)	(Print:Real)	(Fact:Plan)	(Visual:Tactile)	(Target:Line)
1	6	37:30	26:41	49:18	40:27	55:12
2	3	10:8	13:5	10:8	15:3	18:0
3	10	62:53	74:41	46:69	103:12	55:60
4	4	93:80	105:68	103:70	136:37	150:23
5	8	40:27	38:29	55:12	59:8	50:17
6	6	31:34	30:35	42:23	36:29	50:15
7	7	46:49	46:49	76:19	71:24	79:16
8	2	10:11	18:3	19:2	21:0	17:4
9	15	87:105	120:72	110:82	164:28	136:56
10	2	4:5	4:5	5:4	6:3	5:4
11	3	10:11	15:6	15:6	15:6	18:3
12	1	30:31	12:49	56:5	12:49	60:1
13	16	89:95	92:92	137:47	140:44	154:30
14	20	78:107	92:93	168:17	139:46	160:25
15	3	24:28	16:36	37:15	29:23	40:12
16	12	96:106	125:77	126:76	159:43	151:51
17	12	44:77	59:62	94:27	63:58	106:15
Total	130	791:857	885:763	1148:500	1208:440	1304:344

Object, utterance object; Print, 3D-printed liver models; Real, real liver; Fact, fact check; Plan, plan check; Sense, sense used during conversation; Visual, visual check; Tactile, tactile check; Process, surgical process used during conversation; Target, confirmation of the planned resection and/or preservation target; Line, confirmation of the planned and/or ongoing resection line

A comparison of the utterances according to the surgeon’s experience is presented in Table 3. The ratio of the assistant’s utterances during non-expert surgery (54.8%) was significantly more frequent than that during expert surgery (45.7%) (*p* < 0.001). Regarding utterance content, the ratio of fact checks during non-expert surgery (72.3%) was significantly higher than that during expert surgery (63.7%) (*p* < 0.001). The ratios of the senses used during conversation and utterances about the surgical process during conversation were not significantly different between expert and non-expert surgery.

**Table 3:** A comparison of utterance coding according to the surgeon’s experience

	Segments (*n* = 1648)	Surgeon’s experience		
		Expert	Non-expert	*p* value
Utterer				
Operator	791	272	519	<0.001
Assistant	857	229	628	
Utterance object				
3D-printed liver models	885	268	617	0.915
A real liver	763	233	530	
Utterance content				
Fact check	1148	319	829	<0.001
Plan check	500	182	318	
Sense				
Visual check	1208	365	843	0.809
Tactile check	440	136	304	
Process				
Target	1304	388	916	0.292
Line	344	113	231	

Expert, an expert surgeon of hepatobiliary surgery; Non-expert, a surgeon permitted to perform hepatobiliary surgery under an expert’s guidance; Sense, the sense used during conversation; Visual, visual check; Tactile, tactile check; Process, the surgical process used during conversation; Target, confirmation of the planned resection and/or preservation target; Line, confirmation of the planned and/or ongoing resection line

### Conversation analyses

Conversation analyses of the fact and plan checks are presented in Table 4. Utterances associated with 3D-printed liver models used only visual checks without distinction of utterance content. There were no significant differences between the ratio of fact checks concerning the surgical process during conversation in the 3D-printed liver models and that in the real liver (*p* = 0.269). In contrast, the ratio of confirmation of the planned resection line on a real liver (68.0%) was significantly higher than that on the 3D-printed liver models (54.7%) (*p* = 0.003).

**Table 4:** Conversation analyses of fact checking and plan checking through intraoperative reference of 3D print of a liver for thrice or more repeated hepatectomy

	Fact check (1148 segments)				Planned check (500 segments)			
	3D-printed liver model (629 segments)		Real liver (519 segments)		3D-printed liver model (256 segments)		Real liver (244 segments)	
	Target	Line	Target	Line	Target	Line	Target	Line
1	20	0	29	0	2	4	4	8
2	7	0	3	0	6	0	2	0
3	21	8	14	3	16	29	4	20
4	58	0	45	0	36	11	11	12
5	27	5	17	6	4	2	2	4
6	24	0	18	0	4	2	4	13
7	39	0	37	0	2	5	1	11
8	14	2	3	0	0	2	0	0
9	70	0	39	1	15	35	11	21
10	2	0	3	0	0	2	0	2
11	8	1	6	0	4	2	0	0
12	11	0	45	0	1	0	3	1
13	75	0	62	0	5	12	12	18
14	78	8	81	1	1	5	0	11
15	13	0	24	0	3	3	3	9
16	85	2	37	2	17	21	12	26
17	51	0	43	0	3	5	9	10
Total	603	26	504	15	116	140	78	166
*p* value	0.269				0.003			

Target, confirmation of the planned resection and/or preservation target; Line, confirmation of the planned and/or ongoing resection line

A comparison of the conversation analysis according to the surgeon’s experience is shown in Table 5. In 3D-printed liver models, the fact checks concerning confirmation of the planned resection and/or preservation target during non-expert surgery (97.3%) were significantly more frequent than during expert surgery (91.7%) (0.005). In addition, in the real liver, the ratios of non-expert surgery (98.3%) and expert surgery (94.4%) were significantly different (*p* = 0.022). Regarding plan checks, confirmation of the planned resection line on the 3D-printed model during non-expert surgery (64.8%) was significantly more frequent than during expert surgery (41.4%) (*p* < 0.001). In contrast, the ratio of plan checks concerning the surgical process on a real liver was not significantly different between expert and non-expert surgery (0.365).

**Table 5:** A comparison of conversation analyses according to surgeon’s experience

	Segments	Surgeon’s experience				
		Expert		Non-expert		*p* value
Fact check	1148					
3D-printed models	629					
Print—Target	603	143	91.7%	460	97.3%	0.005
Print—Line	26	13	8.3%	13	2.7%	
A real liver	519					
Real—Target	504	153	94.4%	351	98.3%	0.022
Real—Line	15	9	5.6%	6	1.7%	
Plan check	500					
3D-printed models	256					
Print—Target	116	65	58.6%	51	35.2%	<0.001
Print—Line	140	46	41.4%	94	64.8%	
A real liver	244					
Real—Target	78	26	36.6%	52	30.1%	0.365
Real—Line	166	45	63.4%	121	69.9%	

Expert, an expert surgeon of hepatobiliary surgery; Non-expert, a surgeon permitted to perform hepatobiliary surgery under an expert’s guidance; Print—Target, confirmation of the planned resection and/or preservation target on 3D-printed models; Print—Line, confirmation of the planned and/or ongoing resection line on 3D-printed liver models; Real—Target; confirmation of the planned resection and/or preservation target on a real liver; Real—Line, confirmation of the planned and/or ongoing resection line on a real liver

## Discussion

According to a few previous reports, the application of 3D printing technology for living liver transplantation is recognized as a useful and suitable procedure because 3D-printed models facilitate the understanding of spatial relations among anatomical structures [11, 12]. During hepatectomy, 3D angiography reconstructed by MDCT was useful, but visualization was achieved only through a two-dimensional computer screen; therefore, the apprehension of spatial relations among anatomical structures differed among surgeons. In contrast, the application of a 3D-printed liver can indicate the precise spatial relationship among anatomical structures unfailingly [13, 14]. Furthermore, this observation is representable anytime and anywhere [13, 14]. In addition, we previously reported two observations using university non-medical students: (1) these subjects learned faster and inferred the inside of the liver structure more accurately using 3D-printed liver models than 3D liver images; and (2) they were able to identify intrahepatic vascular structures with reference to 3D-printed liver models, the correctness of which equaled that of specialists [23, 24].

In our preliminary experience, a conversation analysis through intraoperative reference of 3D-printed liver models revealed that utilization of the 3D-printed liver models enhanced the construction of elaborate internal mental models of patients’ livers, mental simulation of liver resections, and construction of shared mental models of patients’ livers among doctors [19]. In addition, preoperative conferences using the 3D-printed liver model were convened approximately 1 week before surgery; therefore, shared mental models of patients’ livers among doctors could be constructed with a considerable level of elaboration. In the present study, the mean operative time and blood loss were not significantly different between expert and non-expert surgery because the effects of 3D-printed liver models on the surgeon’s and assistants’ mental models of a real patient’s liver decreased the difference in the apprehension of spatial relationships among anatomical structures between experts and non-experts. In addition, given the significantly more frequent ratio of assistants’ utterances during non-expert surgery than during expert surgery, assistants’ utterances during non-expert surgery are recognized as an effective means of allowing non-experts to perform appropriate surgical processes promptly. The significantly more frequent ratio of fact checks during non-expert surgery than during expert surgery indicates that fact checks are necessary for allowing non-experts to perform appropriate surgical processes. Regarding fact checks, the significantly more frequent ratio of the confirmation of the planned resection and/or preservation target on both 3D-printed liver models and a real liver during non-expert surgery than during expert surgery reveals that the precise apprehension of spatial relationships among anatomical structures is the more important fact check for allowing non-experts to continue the appropriate surgical process than apprehension of the ongoing resection line. Given the significantly more frequent ratio of confirming the planned resection line on 3D-printed liver models during non-expert surgery than during expert surgery, the planning of the resection line is recognized as the more important plan check for allowing non-experts to continue the appropriate surgical process than planning the resection and/or preservation target. By confirming the surgical progress on both 3D-printed liver models and a real liver and performing planning using 3D-printed liver models, complex hepatectomy can be safely performed without depending on the surgeon’s experience.

Regarding the clinical assessment of navigation surgery, there have been some reports on the accuracy of navigation [25–30]. Such an assessment focuses only on topologically navigating accuracy, which should be evaluated using another approach for a clinical assessment. Meanwhile, there have been some reports comparing surgical results between navigation and non-navigation surgery, but such a comparison does not directly demonstrate the clinical utility of navigation surgery [31–34]. Despite the findings described in the proceedings sheet, we found that conversation analyses before, during, and after utilization of 3D-printed liver models were effective assessments of the clinical value that facilitated fostering common recognition among doctors during surgery and continuing surgery safely [21]. This assessment procedure for navigation surgery is quite different from the previously reported assessments [25–34]. In the present study, according to our conversation analysis, the clinical value of 3D-printed liver model navigation was shown to have an educational effect for non-expert surgeons under the guidance of an expert surgeon. Accordingly, a conversation analysis can be applied to other navigation surgeries and is recognized as one of the most important assessment procedures conferring clinical value of navigation surgery.

The major limitation of this study is its limited sample size (*n* = 17) and lack of a control arm. However, conversation analyses included as many as 1648 segments; accordingly, the present results, based on a unique cognitive approach, might have scientific value with reliability. A conversation analysis has been recognized as a useful procedure for analyzing cognitive performance [15–18]. In this study, cognitive performance during 3D-printed liver model navigation was shown to have an educational effect based on conversation analyses. In the future, more detailed conversation analyses during navigation surgery can elucidate the unquantifiable ideation of the surgeon during surgery and elucidate the interrelationship with the development of the artificial intelligence of surgical assist systems.

Another limitation is that before utilization of the 3D-printed liver model navigation, non-expert surgeons had never performed thrice of more repeated hepatectomy; therefore, a comparison of data before and after utilization of the 3D-printed liver model navigation could not be performed.

As we previously reported [13, 14], 3D printing takes approximately 18 h to complete, with the initial liver model costing approximately 50,000 JPY (approximately 335 USD) to make. In addition, the polishing operation requires another 2–3 days. As such, 3D printing technology requires considerable time and money at present. For these reasons, we selected a 3D-printed liver model on a scale of 70%. This downsizing model is easy to handle and practical to use.

Based on the present study, 3D-printed liver models are expected to play an increasingly crucial role in the future education of non-expert surgeons. Before complex hepatectomy, we allow non-expert surgeons to engage in self-teaching with the 3D-printed liver model at the very beginning. Subsequently, thanks to a preoperative conference using the 3D-printed liver model, shared mental models of patients’ livers among the surgical team will be able to be constructed more elaborately. During complex hepatectomy, we will guide non-expert surgeons on performing safe and precise hepatectomy according to the construction of elaborately shared mental models of patients’ livers among doctors using 3D-printed liver models.

In conclusion, the clinical value of 3D-printed liver models is educational for non-expert surgeons who can safely perform complex hepatectomy under the guidance of expert surgeons. A conversation analysis during navigation surgery is an effective assessment procedure for navigation surgery. Further conversation analyses of navigation surgery are interrelated with the development of the artificial intelligence of surgical assist systems depending on the elucidation of the unquantifiable ideation of the surgeon during surgery in the future.
